# Enhancing the Visitor Experience in the Time of COVID 19: The Use of AI Robotics in Pembrokeshire Coastal Pathway

**DOI:** 10.1007/978-3-030-65785-7_55

**Published:** 2020-11-28

**Authors:** Katarzyna Minor, Emmet McLoughlin, Vicky Richards

**Affiliations:** 1 Department for Informatics, Technical University of Munich, Garching bei München, Bayern Germany; 2 Smart Tourism Education Platform (STEP) College of Hotel and Tourism Management, Kyung Hee University, Seoul, Korea (Republic of); 3 Department of Tourism and Service Management, MODUL University Vienna, Vienna, Wien Austria; Welsh Centre for Tourism Research, Cardiff Metropolitan University, Cardiff, Wales UK

**Keywords:** COVID-19, Visitor experience, AI and robotics, Accessibility, Wales

## Abstract

AI and Robots represent a major innovation opportunity for the tourism sector, and their potential impact and application offer several new opportunities to enhance and develop the visitor experience. Nevertheless, there has been limited academic research on the use of robots, together with a limited number of destinations embracing this technology. Focusing on the Pembrokeshire Coastal Path, this research paper outlines how a multi methodological approach could be utilised to examine the use of AI and robotics in helping to enhance the visitor experience during the ongoing COVID-19 pandemic. The researchers anticipate that outcomes from such a study could not only provide theoretical contributions in the area of addressing concerns about accessibility in tourism and leisure settings, but also serve to inform both academia and the wider tourism industry to the benefits such technology can have towards enhancing the visitor experience within social distancing parameters.

## Introduction

The tourism industry has found itself operating in most unprecedented times, where the challenges of yesterday are compounded by the outbreak of global pandemic. This means a lot of destinations, particularly the ones which rely on tourism as a substantial percent of the economic outputs, fight not only the relatively short to mid-term effects related to COVID-19 travel restrictions but also long-term problems of underfunding, seasonal trade variations, attractiveness and inclusivity.

**Fig. 1.**
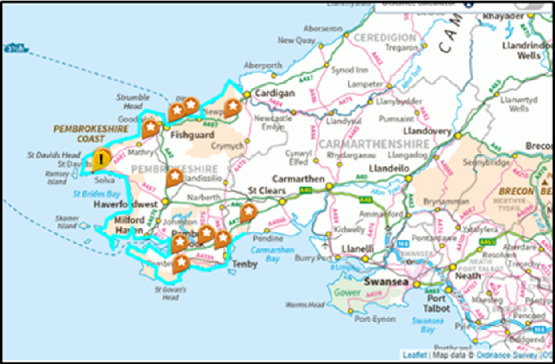
Pembrokeshire Coastal Pathway (Source: National Trails, 2020)

Pre COVID-19, both the Welsh and UK Economies considered tourism as a foundation sector for growth, with the overall tourism value in Wales rising from £4.5bn to £6.3bn in 2017 [4]. Tourism’s impact was never more evident in Pembrokeshire, an area of Wales (see Fig. 1) known for its historical legends, walking trails and international sporting events. Tourism in Pembrokeshire is highly seasonal and generates over 75% of its trade during the months from March to October [4]. Unfortunately, funding for tourism has ‘flatlined’ [4], with the tourism economy being brought to stop due to the COVID-19 pandemic in March 2020. This regrettably coincided with the beginning of the busy tourism season. Thus, the indirect impacts from Brexit funding cuts and over-night closure of all tourism during the pandemic has decimated the local industry which ultimately could take several years to recover, due to both travel restrictions and social distancing regulations [6, 21, 32].

Rebuilding the tourism trade in post pandemic world may be problematic for Pembrokeshire and Wales in general, as 16% of domestic visitors to Wales have identified themselves as having a long-term illness or disability, and further 28% of domestic visitors are also in the age bracket of 55 plus [31]. Wales as a destination is highly reliant on domestic tourism, this puts over a quarter of domestic tourists’ arrivals in the ‘at-risk category’, therefore, less likely to go on holiday. This puts additional pressure on destinations to reopen and operate with an increased safety and social distancing as an underlying principle in order to enable all possible tourist groups a safe return and enjoyment of Welsh countryside.

One of the ways to enable the safe tourism interactions is introduction of AI and robotics to the day-to-day operations. Indeed many countries, such as the Netherlands and Singapore amongst others, have successfully utilised robots in hospitality and healthcare settings with the aim to reduce human to human contact, thus, helping to both mitigate the spread of the virus and as a means to getting back to ‘normal’ [33]. This conceptual research paper is aimed to bring together Universal Design principles, robotics and novel tourism visitor attractions together in one research study. Central to it is the use of purposely coded, humanoid robot tourist guides, which will be placed along Pembrokeshire Coastal path (see Fig. 1). It is anticipated that this study will contribute to the body of knowledge in the areas of the utilisation of Accessibility to All in robot design, empirical evidence of tourist – robot interactions and enhancement of visitor experience, as well as the effectiveness of robots to support social distancing requirements.

## Research Background

This anticipated study comes at a time when the tourism and leisure industry in Pembrokeshire is primarily made up from micro and small enterprises, with visitor economy contributing £585m annually [4]. Whilst the region’s main strategy is to focus value in opposition to volume of tourists, there are growing calls from the Welsh Government “to be putting our energies into thinking innovatively for the future” [17]. Pembrokeshire as a destination, therefore, aims to maximise behaviour, wellbeing, health and the use of technology as strategic goals for 2020–2025. Thus, the use of latest technologies, such as AI and robots could tie in with the overall goal of enhancement of experience [4]. AI and Robots represent a major innovation opportunity for the tourism sector, and their potential impact and use may offer many new avenues to enhance and develop the visitor experience [2, 16, 26].

Pembrokeshire as a destination identified its main weakness as fragmented marketing, characterised by lack of joint ‘Pembrokeshire narrative’ and uncoordinated information provision. Pembrokeshire’s fragmented tourism industry makes it difficult to coordinate those marketing efforts, especially considering lack of external funding. Particularly, under current economic climate the businesses are likely to concentrate on strategies of survival and safe operations, rather than long-term strategic destination management efforts. Yet, according to [22] AI has now entered the tourism and hospitality setting and is being consumed by both organisations and destinations in order to gain a competitive advantage, in what is a highly dynamic market. Indeed, [29, 30] theorise that the future of the sector is likely to be dominated by automated and robotic smart solutions, particularly robot use for information-giving functions has been reported as welcome [8, 29]. Therefore, utilisation of Robot tourist guides would enable the creation of sense of place by providing a robust, customised and coordinated tourist information fitting with the ‘Pembrokeshire narrative’ of a place of natural beauty and rich cultural heritage.

Both [7] and later [20] argue that tourist experiences are reflective and inherently personal, there is a need to provide opportunities to travellers to interact with the technology. This spans from the belief that travel often is considered a sense-making process whereby traveller’s construct the touristic experience by learning, understanding, and feeling the places visited [11, 28]. In other words, lack of real time experience of robots in natural tourist setting is likely to affect the attitudes and perceptions of this technology. Indeed, [12] point out not a lot of travellers have had actual interactions with robots and the existing research to date does not typically use actual interactions between humans and robots for the basis of the studies; majority of research is either conceptual (e.g. [14]), engineering (e.g. [5]), experimental (e.g. [18]) or survey based (e.g. [9]). Yet, despite its increasing importance, there has been limited academic research on the use of robots in tourism, and even fewer empirical research focusing on travel interactions with robots. In fact, [10] note, in their literature review of 131 tourism-related papers, just over half of those focused on the adaptions of robots by companies, with less attention paid to human-robot interactions. Furthermore, restaurants, hotels, airports and bars dominated the sector focus of the reviewed studies, indicating relative lack of broader tourism application of robotics. This may be caused by only a few destinations embracing this technology, mostly concentrated in Asia and commercialisation of the technologies in places such as Hen na Hotel in Nagasaki or FlyZoo Hotel in Hangzhou. This in part may be explained by a greater acceptance of automation rooted in Eastern cultures when compared with Western cultures [3].

Last consideration should be given to the tourist base of the area. The focus here is two-fold- on actual and potential tourist base for the region. First, as identified over a quarter of tourists are identified as ‘at risk’ due to COVID- 19; as people must avoid physical interaction, [23] argue that service robots can be a useful tool to ensure a high level of physical social distancing. Therefore, the use of robotics could potentially mean human to human contact frequency could be reduced, while also ensuring the recommended social distancing is maintained. However, more research is required to help determine how AI and robotics can help contribute to enhancing the visitor experience in tourism setting, particularly now in the time of COVID-19.

Second, existing research conducted by Visit England in 2018 suggests that 430,000 British adults with disabilities did not take a domestic holiday as a result of accessibility issues [27]. Despite legal requirements set out in the UK through the Equality Act (2010), the industry is still working towards equal participation for disabled people. Thus, whilst the industry talks of the value of the ‘purple pound’ (disabled people); disabled citizens still encounter barriers to their participation in tourism experiences as amongst other areas of life. It is anticipated that this study will help in addressing many of the concerns about accessibility in tourism and leisure settings and bias issues related to technologies and AI. This is in line with [24] observations that technology and visitor information alike must be accessible to people with a wide range of disabilities, as well as Welsh Government policies aimed at accessibility to all. As [27] research argues that the industry is missing out on a potential £116.7 million as a result of inaccessible infrastructure, the accessible nature of the project creates an opportunity for the Pembrokeshire destination to tap into new tourist markets, thus providing a much-needed economic advantage.

## Proposed Methodology

In order to examine the use of AI and robotics to help enhance the visitor experience during a time of COVID-19, this research paper suggests a multi method approach, thus having the benefit of combining different approaches and research methods [13, 19]. The researchers therefore assume a three -stage study: 1) development, 2) implementations, 3) evaluation, and envisages a use of 10–15 small humanoid robots, positioned along the Pembrokeshire coastal path.

Accessibility for all is embedded into the project as a whole- from conception to the execution, by ensuring the technology as well as the information are accessible to people with a wide range of disabilities. This will be achieved by consulting different stakeholder groups, to include disability groups, with regards to functionalities of the robots, formats of information and type of information which the robots should have to maximise the benefit and access to all.

In stage one of the studies the researchers suggest a series of focus groups with four distinct groups (visitors, tourism organisations, visitors identifying as having a disability and a mixed group consisting of all three) to determine how their visitor experiences could be enhanced. Since focus groups are ‘particularly useful for exploratory research where rather little is known about the phenomenon of interest’ [26:15] the aim here would be to stimulate some discussions with a use of a pilot robot. The findings will inform the development and design of the specific functionalities of the robots.

It is suggested that research on enhancing the visitor experience through the use of AI should use humanoid robots, in line with [34] and [15] study recommending human-like behaviours to be programmed onto the robots for greater acceptance. Whilst [25] note that the physical form of the robot does not affect the trust towards them; [34] found that the ‘gender’ of the robot is likely to affect acceptance of it. Furthermore, since [35] highlight that, in line with Media Equation theory, people tend to interact with technology in a social way. Therefore, researchers would need to have a variety of robot-personas developed to account for all possible outcomes and maximise the potential of positive interactions.

Implementation stage would naturally involve the installation of robots in tourist hot spots, such as check-in desks, cafes, tourist information centres, and other places of tourist interest where access to secure WIFI can be obtained. It is envisioned that this is where the robots will supply, when promoted, specific information to visitors and walkers. A variety of suitable methods could be employed during this stage, from direct observations by the research team, pre- and post- encounter interviews (to gauge emotions [35]), and data observed through the robots (e.g. facial expressions/movement, types of interactions, commands used, information requested, length of interactions, distance, ease of use [35]). Therefore, the researchers argue that such characteristics could contribute to creating a unique and safe visitor experience.

A significant aspect of such a project would be ongoing consultation and collaboration with relevant stakeholders. Such an approach would be invaluable to help grow the interaction with the local stakeholder base. Furthermore, the businesses where robots were to be located would need to be consulted with the regards to the perceived usefulness and drawbacks of having this technology on the premises.

The researchers acknowledge that such research carries substantial ethical implications which need to be addressed. For example, as visual data would need to be generated to analyse the interactions, the researchers note the need for a carefully considered consent process to be implemented. Furthermore, all interactions recorded without the necessary consent of the participants will have to be removed and all other recordings would need be stored securely in line with current GDPR guidelines. Since such technology would be operated remotely using existing WIFI infrastructure, there is a potential risk of hacking and security. Other potential limitations and ethical considerations acknowledged by the researcher would relate to robot capabilities and design. For example, the robots cannot detect and interpret human emotions, therefore, will not be able to deal with and react to anger or frustration, thus there will be a need for people to deal with enquires in these situations. Furthermore, such robots would not have the ability to prioritise if faced with multiple requests/interactions at the same time, as such this could lead to frustration and anger [1].

## Concluding Remarks

Artificial Intelligence and technology have helped to revolutionise various industries around the world [22]. And such innovative inventions are needed now more than ever when it comes to enhancing the Visitor Experience in the time of COVID 19. Therefore, this research paper outlines a methodological proposal to analyse how the use of AI robotics along the Pembrokeshire Coastal Pathway could help contribute to greater levels of visitor satisfaction by providing a unique and novel experience. The main theoretical contributions of this proposed study include addressing the concerns about accessibility in tourism and leisure settings, where the researchers have prioritised addressing these issues in developing the robot technology, thus, making the methodology unique and original.

Furthermore, it is envisaged that findings of such a proposed study could also be used to inform both academia and industry with regards to the potential of the use of purposely designed, accessible, AI powered technologies to maximise visitor experience within social distancing setting. As such a project would be the first to examine the feasibility of incorporating AI technology to help contribute to greater levels of visitor satisfaction as a result of COVID 19, it should be noted that there is limited research to draw comparisons. Therefore, data generated could enable the improved future design of technology and maximise the benefits it can offer. Such baseline information will benefit and inform future long-term studies around what type of interactions are desirable by all visitors, what type of information is mostly requested. Particularly it will inform to what extent the use of technology minimises the staff’s exposure when faced with frequently asked questions, thus, highlighting the potential of creating safe working environment for tourism-related businesses.
